# Trends in hip and knee replacement length of stay and patient demographics in England: a population-based study of 1,455,842 primary procedures

**DOI:** 10.1186/s12916-025-04294-6

**Published:** 2025-10-14

**Authors:** Jonathan M R French, Kevin Deere, Adrian Sayers, Michael R Whitehouse

**Affiliations:** 1 Musculoskeletal Research Unit, Learning and Research Building, Southmead Hospital, University of Bristol Medical School, Bristol, BS7 0UL United Kingdom; 2 National Institute for Health Research Bristol Biomedical Research Centre, University Hospitals Bristol and Weston NHS Foundation Trust and University of Bristol, Bristol, United Kingdom

**Keywords:** Length of stay, Inpatient stay, Hospital stay, Trends, Hip replacement, Knee replacement, Joint replacement, Arthroplasty, Characteristics, Demographics

## Abstract

**Background:**

Length of stay (LOS) after hip and knee replacement has decreased steadily in the modern era with enhanced recovery protocols, enabling healthcare systems to address rising surgical demand in an ageing, comorbid population. This study examines trends in LOS, patient characteristics, and their associations for NHS-funded procedures in England, covering a period that includes the COVID-19 pandemic.

**Methods:**

Data from the National Joint Registry and Hospital Episode Statistics were linked to identify patients who underwent primary total hip replacement (THR) and total or unicompartmental (partial) knee replacement (TKR/UKR) in England between January 2010 and March 2022. LOS was analysed using flexible parametric models to estimate median values over time, with subsequent adjustment to examine associations between changing patient characteristics. Trends in 30-day readmission rates were also analysed.

**Results:**

From 2010 to 2022, median LOS decreased from 4.26 days (95% CI 4.22 to 4.30) to 2.75 days (95% CI 2.74 to 2.77) for THR, from 4.35 days (95% CI 4.32 to 4.39) to 2.91 days (95% CI 2.90 to 2.92) for TKR, and from 3.2 days (95% CI 3.16 to 3.25) to 1.91 days (95% CI 1.89 to 1.95) for UKR. Variability also decreased. There were no increases in crude 30-day readmission rates. Trends in patient demographics showed increasing comorbidity, obesity, male sex, affluence, and use of the independent sector, all of which were associated with LOS and had the overall effect of slightly attenuating its reduction. Significant changes in patient characteristics occurred around the time of the COVID-19 pandemic but have since resumed previous trends.

**Conclusions:**

Patients in England now typically stay fewer than three days for total hip or knee replacement and under two days for partial knee replacement. Despite demographic trends towards characteristics associated with longer LOS, reductions have occurred independently of these changes, suggesting potential for further shortening. However, as these diverging trends continue, ensuring equitable access to surgery will be increasingly important.

**Supplementary Information:**

The online version contains supplementary material available at 10.1186/s12916-025-04294-6.

## Background

Hip and knee replacements are common and successful operations for treating end-stage arthritis [1, 2]. Globally, over two million hip and knee replacements are performed per year [3, 4], including approximately 200,000 annually in England [5]. This incidence is projected to rise significantly as populations age [6]. This burden has been further compounded by the effects of the COVID-19 pandemic, where disruption to routine hospital services incurred substantial losses of joint replacement activity [7].

Meeting the rising demand for joint replacements will require either increased resources or reductions in procedural costs and resource use. One of the major modifiable components of the cost of joint replacements is the LOS in hospital [8], and reducing it without compromising on patient outcomes is therefore a potential solution to the challenge of provision.

In the last few decades, the length of stay (LOS) after joint replacement has decreased substantially [8]. This reduction is largely due to several factors, including standardised Enhanced Recovery After Surgery (ERAS) protocols, earlier postoperative mobilisation, better patient education, and advancements in pain management [9, 10]. Previous estimates based on data from England were that the mean LOS for total hip and knee replacement fell from more than 14 days in 1997 to around 5 days in 2014 [8]. In the near future, there are initiatives for day-case surgery, i.e. a LOS of zero days, to become standard practice for joint replacement [11–13].

Conversely, both the general UK and patient population undergoing joint replacement are increasing in age, morbidity, and obesity [14, 15], all factors associated with longer lengths of stay following joint replacement [16, 17]. This dichotomy has not been explored in detail. To date, there has been no national study examining LOS trends in England, nor any that have analysed changes in patient characteristics around the time of the COVID-19 pandemic. Similarly, no previous national research has assessed the relationship between demographic trends and LOS at the population level. Understanding these patterns is important for evaluating the feasibility and implications of further reductions in LOS. Optimising peri-operative pathways was a James Lind Alliance Priority Setting Partnership top 10 priority area highlighted by patients, carers, and clinicians in 2014 [18].

This study aimed to address these evidence gaps by describing contemporary trends in LOS following NHS-funded total hip replacement (THR), total knee replacement (TKR), and unicompartmental knee replacement (UKR) in England, using routinely collected national data. We aimed to examine trends in patient characteristics and assess their potential impact on observed changes in LOS.

## Methods

### Study design and data sources

**Fig. 1:**
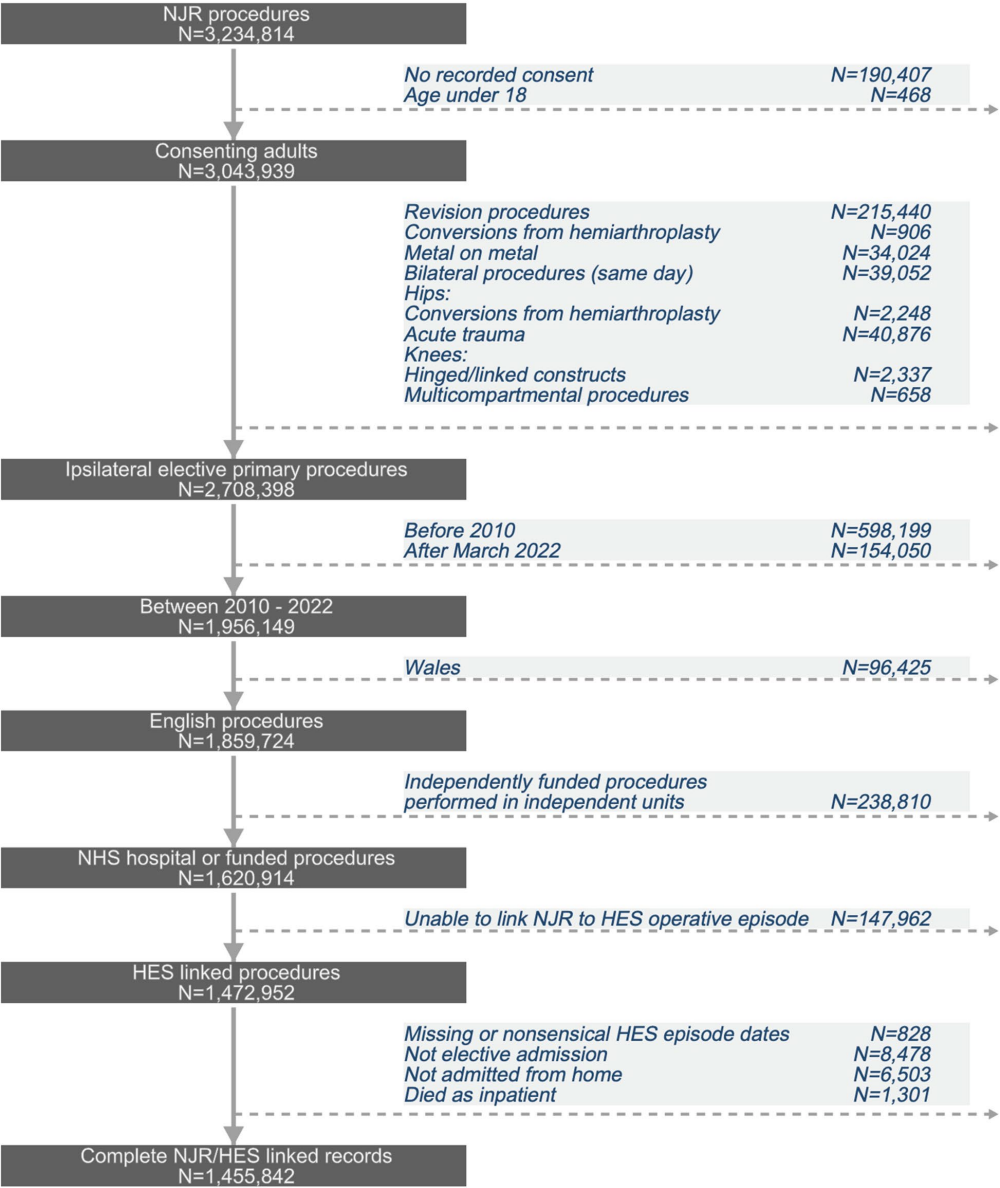
Data flowchart for linked National Joint Registry (NJR) and Hospital Episode Statistics (HES) hip and knee replacements

This was a population-based prospective cohort study using linked, routinely collected data for NHS-funded hip and knee replacements undertaken at both NHS and independent hospitals in England from 1st January 2010 to 31st March 2022. Data from the National Joint Registry (NJR) of England were linked to the NHS Hospital Episode Statistics (HES). Data submission to the NJR is mandatory for all hip and knee replacements and includes patient, surgeon, and operation details. Annual data quality audits consistently show > 96% completeness of all primary hip and knee data [19]. HES records all NHS-funded activity in England and is used for the accurate reimbursement of NHS providers for their activities. For the purposes of this study, HES episodes required cleaning into ‘superspells’ [20] using the process outlined in Additional file 1: Figure S1. HES linkage was achieved in 90.9% of procedures (Fig. 1).

Three groups of operations were considered separately: total hip replacement (THR), total knee replacement (TKR), and unicompartmental knee replacement (UKR). Trends in LOS and patient characteristics were initially assessed using univariable models. LOS was then adjusted for patient characteristics in multivariable models to examine the association between observed LOS trends and demographic changes.

### Selection criteria

All patients aged 18 years and older having an elective primary hip or knee replacement were eligible for inclusion. Exclusion criteria were lack of NJR patient consent, reason for surgery recorded as acute trauma or cancer, same-day bilateral procedures, missing or non-sensical HES episode data, non-elective admissions, patients not admitted from home, and patients who died in hospital (Fig. 1).

### Variables

The exposure was the date of surgery. The outcome was LOS, defined in calendar days as the discharge date minus the admission date, obtained from HES. Index of Multiple Deprivation (IMD) quintiles, a measure of socioeconomic status (with IMD quintile 5 implying the highest level of deprivation), were also derived from HES. Covariates obtained from NJR were age, sex, American Society of Anaesthesiologists (ASA) grade, and hospital sector. Body mass index (BMI) was missing in 22.2% of NJR cases and was therefore converted to binary obesity with a cutoff of ≥ 30 kg/m^2^, with missing values imputed using presence or absence of obesity (E66) ICD-10 (International Classification of Diseases, 10th revision) codes from HES. Unplanned hospital readmissions within 30 days of discharge were analysed, defined using emergency HES admission method codes (Additional file 1: Fig. S1).

### Statistical analysis

LOS data is strongly positively skewed, and no universally accepted method exists for its statistical modelling [21]. Using the median rather than the mean has the advantage of reducing the impact of outliers and is preferable for skewed distributions. We therefore treated LOS as time-to-event data and conducted a survival analysis using flexible parametric modelling to generate median estimates over time for each procedure, which also allowed for subsequent statistical adjustment. Day case surgery, i.e. LOS 0 was imputed as 0.1. Restricted cubic splines were used to model the baseline hazard function of discharge, with the best fitting and most parsimonious unadjusted model selected using the Bayesian Information Criterion (BIC) and visual inspection of predicted survival curves against observed Kaplan–Meier estimates (Additional file 1: Fig. S2). Date of surgery was modelled using restricted cubic splines to account for non-linearity. This approach enabled visualisation of trends in a continuous, time-varying manner whilst also allowing for point estimates of median LOS at the start and end of the study period.

As the relationship between LOS and operation date was found to be generally linear, to aid subsequent comparison of the overall effect, simpler flexible parametric models were fitted with time modelled using year of surgery as a continuous variable. The estimated effect of year of surgery on LOS was compared both with and without adjustment for patient characteristics to explore how the association with time was modified by demographic changes. This generated hazard ratios (HR) for discharge, with a non-intuitive interpretation of HRs above 1 representing an association with a higher hazard of discharge, i.e. a shorter LOS, and HRs below 1 representing an association with a lower hazard of discharge, i.e. a longer LOS. The following covariates were examined in the multivariable models: age (restricted cubic splines), sex (female/male), ASA (I, II, III, IV–V), obesity (yes/no), IMD quintile (1 to 5), and hospital sector (NHS/independent). These were selected based on established clinical relevance [22] and prior literature identifying factors known to influence LOS [16, 17]. Flexible parametric models were fitted in the same manner as the univariable models initially, with model fit for the baseline hazard function assessed using the BIC and visual inspection of plots. Covariates were then added sequentially, and improvements in model fit evaluated using likelihood ratio tests.

Trends in patient characteristics and 30-day readmissions for each type of joint replacement were described using univariable linear regression models including year of surgery and the above dichotomised covariates. Estimates were visualised against observed rolling monthly means centred across one year, with 95% confidence intervals for both.

#### Missing data and sensitivity analyses

IMD data was missing in 0.73% of cases. The characteristics of these were inspected and found to be largely comparable, aside from having a larger proportion undertaken in NHS (public sector) hospitals (Additional file 1: Table S1). These records had otherwise complete data and were therefore included in univariable models in order to contribute towards LOS trend data, but excluded from the multivariable models. A sensitivity analysis was performed to test for any difference in univariable model results if they were also excluded from these.

Median is not always preferable to mean for interpreting skewed data, and means are often quoted in the literature [17]. Therefore, a sensitivity analysis using linear regression in the same manner as the flexible parametric survival analysis was performed. Histograms showing the distribution of LOS, with estimated medians and means from both analyses, can be seen in Additional file 1: Fig. S3. All analyses were performed using Stata (version 18, StataCorp, USA).

### Role of the funding source

The funders had no role in study design, data collection, data analysis, data interpretation, or writing of the report.

## Results

The study population comprised 1,455,842 procedures of which 668,615 were THR, 715,938 were TKR, and 71,289 were UKR. A summary of patient characteristics is displayed in Table 1.

**Table 1:** Summary of patient characteristics. Numbers in brackets represent standard deviation for continuous variables and percentages for categorical variables. Abbreviations: *ASA*, American Society of Anaesthesiologists; *IMD*, Index of Multiple Deprivation; *NHS*, National Health Service

	**Total hip replacement** (*N* = 668,615)	**Total knee replacement** (*N* = 715,938)	**Unicompartmental knee replacement** (*N* = 71,289)
Age	68.34 (11.26)	69.66 (9.23)	63.32 (10.19)
Sex			
Female	398,873 (59.7%)	409,869 (57.2%)	35,153 (49.3%)
Male	269,742 (40.3%)	306,069 (42.8%)	36,136 (50.7%)
ASA grade			
I—Fit and healthy	80,619 (12.1%)	54,540 (7.6%)	11,803 (16.6%)
II—Mild disease not incapacitating	462,954 (69.2%)	522,194 (72.9%)	52,078 (73.1%)
III—Incapacitating systemic disease	121,756 (18.2%)	136,917 (19.1%)	7337 (10.3%)
IV-V—Life threatening disease	3286 (0.5%)	2287 (0.3%)	71 (0.1%)
Obesity	253,482 (37.9%)	374,754 (52.3%)	35,271 (49.5%)
IMD quantile			
Least deprived	159,663 (23.9%)	154,669 (21.6%)	18,872 (26.5%)
2	163,495 (24.5%)	165,678 (23.1%)	17,085 (24.0%)
3	147,304 (22.0%)	156,992 (21.9%)	15,386 (21.6%)
4	112,335 (16.8%)	129,918 (18.1%)	11,482 (16.1%)
Most deprived	80,222 (12.0%)	104,083 (14.5%)	8005 (11.2%)
Missing	5596 (0.8%)	4598 (0.6%)	459 (0.6%)
Unit sector			
NHS	470,874 (70.4%)	499,903 (69.8%)	46,748 (65.6%)
Independent	197,741 (29.6%)	216,035 (30.2%)	24,541 (34.4%)
NHS funded	661,344 (98.9%)	711,643 (99.4%)	70,754 (99.2%)

**Fig. 2:**
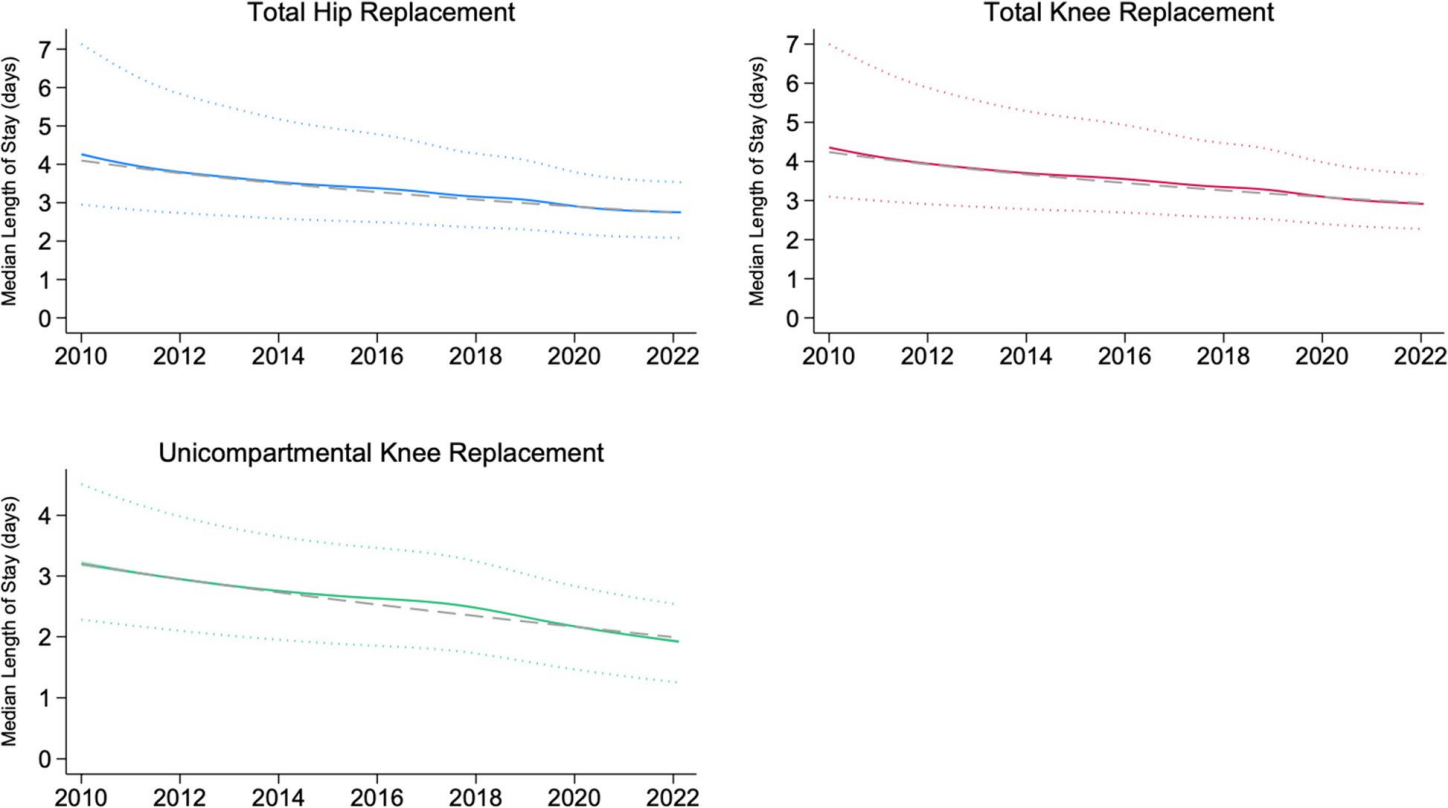
Trends in median LOS over time, generated using restricted cubic splines for operation dates in flexible parametric survival models to account for non-linearity (solid coloured lines). 95% CIs are represented by shaded areas around these lines, which are mostly too narrow to be visualised. Dotted coloured lines represent interquartile ranges. Grey dashed lines represent linear estimates; these models were used for subsequent adjustment in order to evaluate associations between demographic changes and LOS

The LOS for all procedures significantly reduced over time in a broadly linear fashion (Fig. 2). For THR, estimated median LOS was 4.26 days (95% CI 4.22 to 4.30) in 2010 and 2.75 (95% CI 2.74 to 2.77) in 2022. For TKR, estimated median LOS was 4.35 days (95% CI 4.32 to 4.39) in 2010 and 2.91 (95% CI 2.90 to 2.92) in 2022. For UKR, estimated median LOS was 3.20 days (95% CI 3.16 to 3.25) in 2010 and 1.91 (95% CI 1.89 to 1.95) in 2022. Variability reduced over time, demonstrated by the IQR narrowing significantly for each procedure (Fig. 2). Observed yearly values are presented in Additional file 1: Table S2.

The overall linear trend for each procedure was a reduction in median LOS per year of 0.11 days (HR 1.089, 95% CI 1.088 to 1.089) for THR, 0.106 days (HR 1.087, 95% CI 1.086 to 1.087) for TKR, and 0.099 days (HR 1.105, 95% CI 1.102 to 1.108) for UKR. When adjusted for changes in patient characteristics, there were small increases in the magnitude of the effect, indicating that these trends have occurred despite the evolving demographics (Table 2).

**Table 2:** Unadjusted and adjusted flexible parametric models examining the association between year of surgery and LOS, to determine the extent to which trends could be explained by changes in patient characteristics. A HR of more than 1 indicates a higher hazard of discharge, i.e. associated with a shorter LOS, and vice versa for HRs of below 1. Note age had a non-linear association with LOS and therefore individual HRs for each spline cannot be directly interpreted; see Additional file 1: Fig. S4. Abbreviations: *HR*, hazard ratio; *CI*, confidence interval; spline, restricted cubic spline; *ASA*, American Society of Anaesthesiologists grade; *IMD*, index of multiple deprivation

	**Total hip replacement** **(HR (95% CI)**		**Total knee replacement** **(HR (95% CI)**		**Unicompartmental knee replacement (HR (95% CI)**	
	Unadjusted	Adjusted	Unadjusted	Adjusted	Unadjusted	Adjusted
Year	1.089	1.106	1.087	1.096	1.105	1.111
	(1.088 to 1.090)	(1.105 to 1.107)	(1.086 to 1.088)	(1.096 to 1.097)	(1.102 to 1.108)	(1.108 to 1.113)
Age spline 1		0.773		0.795		0.911
		(0.771 to 0.775)		(0.793 to 0.797)		(0.904 to 0.919)
Age spline 2		1.178		1.125		1.107
		(1.175 to 1.181)		(1.122 to 1.128)		(1.099 to 1.115)
Age spline 3		1.017		1.003		1.031
		(1.015 to 1.020)		(1.001 to 1.006)		(1.024 to 1.039)
Sex: Male		1.200		1.130		1.210
		(1.194 to 1.206)		(1.125 to 1.136)		(1.192 to 1.228)
ASA: 1		1		1		1
		(ref)		(ref)		(ref)
ASA: 2		0.850		0.863		0.909
		(0.843 to 0.856)		(0.855 to 0.871)		(0.890 to 0.928)
ASA: 3		0.544		0.592		0.652
		(0.538 to 0.549)		(0.586 to 0.598)		(0.632 to 0.673)
ASA: 4–5		0.353		0.402		0.293
		(0.341 to 0.366)		(0.385 to 0.420)		(0.231 to 0.371)
Obesity		0.945		0.962		0.951
		(0.940 to 0.950)		(0.958 to 0.967)		(0.936 to 0.965)
IMD quintile: 1 (least deprived)		1		1		1
		(ref)		(ref)		(ref)
IMD quintile: 2		1.002		1.003		1.008
		(0.995 to 1.009)		(0.996 to 1.010)		(0.987 to 1.029)
IMD quintile: 3		0.983		0.977		0.988
		(0.976 to 0.990)		(0.970 to 0.984)		(0.968 to 1.010)
IMD quintile: 4		0.936		0.945		0.950
		(0.929 to 0.943)		(0.938 to 0.952)		(0.928 to 0.972)
IMD quintile: 5 (most deprived)		0.871		0.911		0.909
		(0.864 to 0.879)		(0.904 to 0.918)		(0.885 to 0.933)
Independent sector		1.662		1.859		1.357
		(1.653 to 1.671)		(1.849 to 1.869)		(1.335 to 1.379)

Factors associated with significantly longer LOS were female sex, higher ASA grade, obesity, and higher deprivation (Table 2). Procedures undertaken in independent sector hospitals were associated with significantly shorter LOS. The relationship between age and LOS was non-linear, but generally, advancing age was associated with longer LOS (Additional file 1: Fig. S4).

**Fig. 3:**
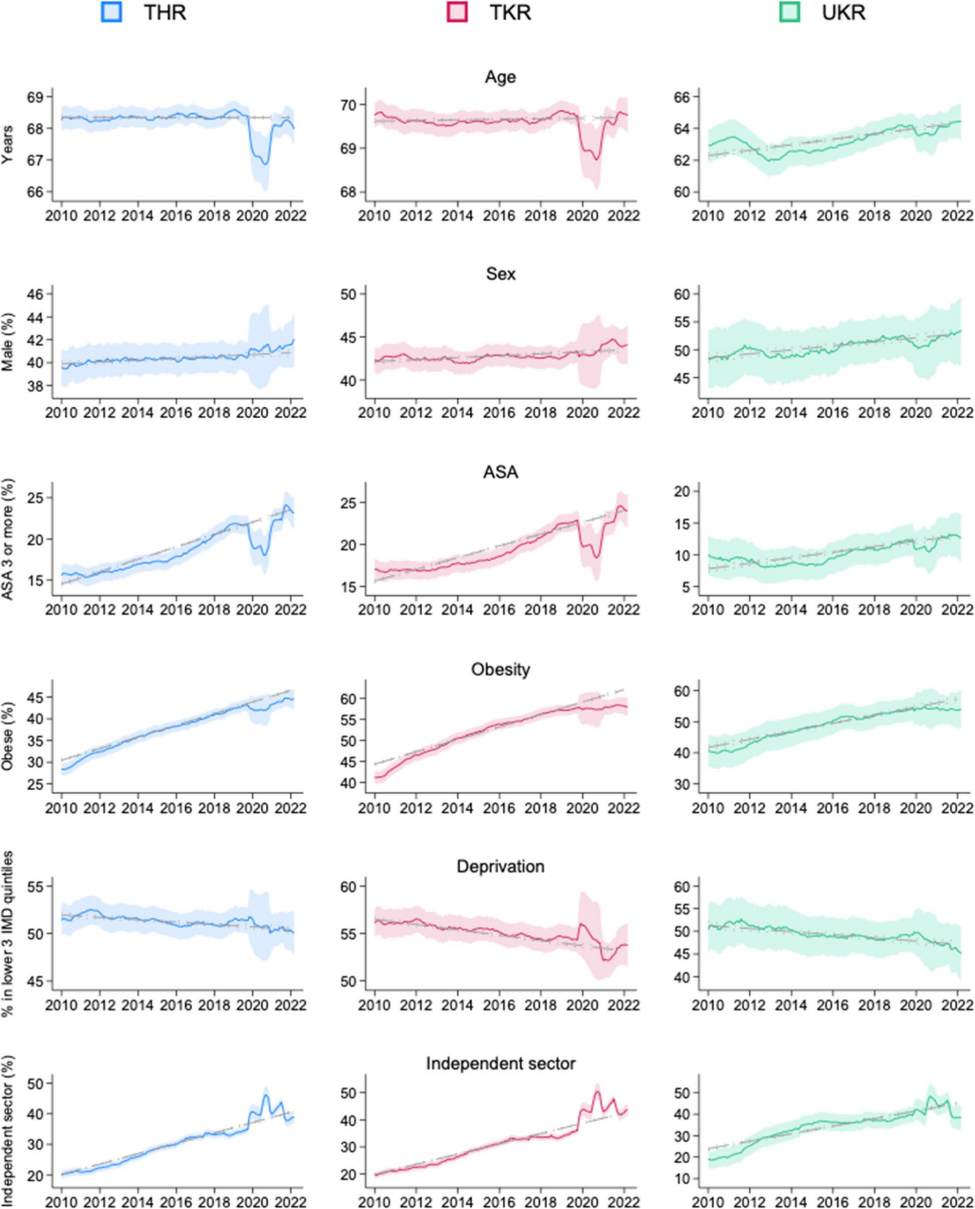
Trends in patient characteristics over time, by procedure. The solid lines represent rolling monthly means, centred across 12 months, with the shaded area representing 95% CIs. The grey dashed line represents linear regression output, with dots representing 95% Cis

Trends in average patient characteristics for each procedure are displayed in Fig. 3 and Additional file 1: Table S3. All procedures demonstrated increases in patient comorbidity (proportion of patients with an ASA grade of three or higher), obesity, male sex, and procedures performed in independent hospitals. Small decreases in relative deprivation were observed. Patient age increased for UKR but did not significantly change for THR or TKR. Disruptions were seen in all characteristics from 2020, around the time of the COVID-19 pandemic.

**Fig. 4:**
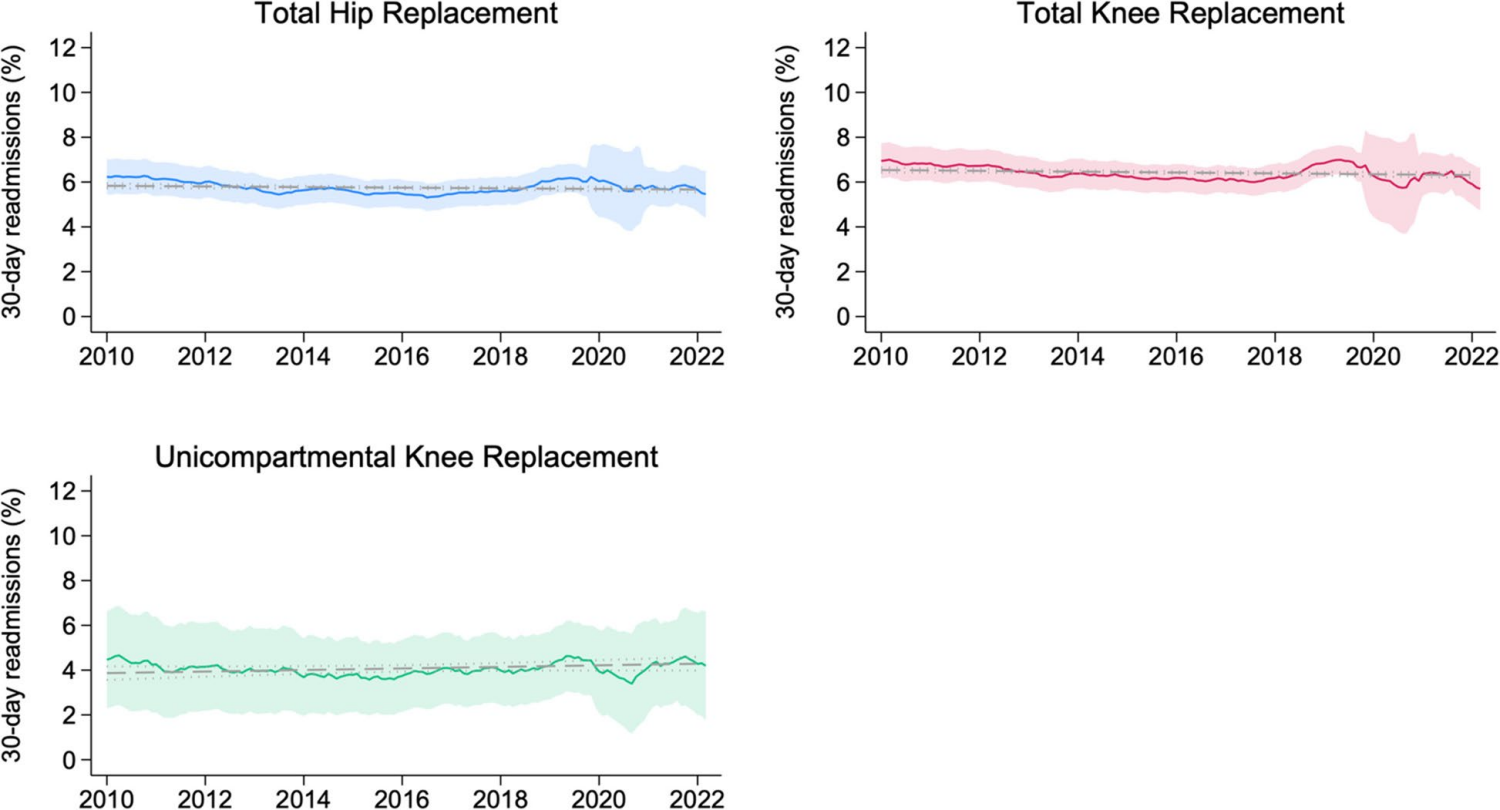
Trends in 30-day readmissions to hospital, by procedure. There were no increases in readmission rates over time. The solid lines represent rolling monthly means, centred across 12 months, with the shaded area representing 95% CIs. The grey dashed line represents linear regression output, with dots representing 95% CIs

There were no increases in rates of unplanned hospital readmissions within 30 days of discharge (Fig. 4). Trends showed no significant change over time for THR (− 0.014% per year, 95% CI − 0.031 to 0.003) or UKR (0.034% per year, 95% CI − 0.009 to 0.079), but a significant decrease in 30-day readmissions for TKR (− 0.019% per year, 95% CI − 0.036 to −0.001).

Sensitivity analyses showed no difference in results when cases missing IMD data were excluded from univariable models (Additional file 1: Table S4). Sensitivity analyses using linear regression to generate mean values produced similar trends to the median values in the main analysis, though with a greater absolute reduction in estimated LOS, likely due to the greater impact of reduced variability on means (Additional file 1: Table S2, Table S5, Fig. S5).

## Discussion

This population-based study used routinely collected national data to examine trends in LOS and patient characteristics for 1,455,842 NHS-funded primary hip and knee replacements in England. Between 2010 and 2022, the median LOS decreased from 4.26 to 2.75 days for total hip replacements, from 4.35 days to 2.91 days for total knee replacements, and from 3.2 days to 1.91 days for unicompartmental knee replacements. The variability in LOS also decreased. Over this period, there were no concurrent increases in crude 30-day readmission rates. Trends in patient demographics showed increasing comorbidity, obesity, male sex, affluence, and use of the independent sector, all of which are significantly associated with LOS. However, the observed trends in shorter hospital stays have continued despite these demographic changes.

This is the first study to examine national trends in LOS in England. The study period continues from that of a previously reported regional subset of 21,221 patients in England between 1997 and 2014, where mean LOS for total hip and knee replacement fell from over 14 to 5 days, albeit without demographic change over time [8]. Similarly, Scottish Arthroplasty Project (SAP) data show a decrease in median LOS from a median of 5 days in 2010 to 2 days in 2023 [23]. Internationally, LOS is comparatively shorter in the USA, where American Joint Replacement Registry (AJRR) data reports the mean falling from around 3 days in 2012 to 1.2 in 2022 [24], and Canada, where the Canadian Joint Replacement Registry (CJRR) reported a median of 2 days for 2021 [25]; however, unlike the present study, these registries do not have national coverage which potentially makes them less representative [19]. A registry study from Denmark of 166,833 procedures with national coverage reported that median LOS had fallen to 1 by 2020 [26]. Other published multi-centre studies from Europe, Asia, and Australasia generally report longer lengths of stays than this, ranging from approximately 4 days in Finland [27] and New Zealand [28] to above 20 in Japan [29] (see table of studies in Additional file 1: Table S6 [8, 23–40]. This study is one of the few in the literature using a dataset with national coverage, ranks among the largest to date, and is the only one of its kind from England.

Regarding risk factors for prolonged LOS, a 2015 systematic review and meta-analysis on 14 studies on THR reported strong associations for higher ASA grade, number of comorbidities, and presence of heart or lung disease [16]. Similarly, for TKR, a 2019 meta-analysis of 68 studies identified increased age, female sex, obesity, non-white ethnicity, an ASA grade of III or more, and anaemia as risk factors. The latter study observed a mean reduction in LOS in published studies of 3.97 days per decade since the 1980s [17]. Previous individual UK-specific studies are in concordance with the present study having also identified female sex, increasing age, deprivation, and comorbidity as risk factors for prolonged LOS [41, 42]. Most patient characteristics associated with longer lengths of stay therefore appear consistent across different countries.

After adjusting for these known predictors of LOS, it was found that LOS has continued to decline despite an increasingly frail and obese patient cohort. This suggests broader changes in care delivery are driving these reductions, which likely include the widespread adoption of enhanced recovery after surgery (ERAS) protocols, greater emphasis on patient education, evolving expectations among patients and staff, and improvements in postoperative rehabilitation [9, 10]. Advances in anaesthetic techniques and the use of multimodal, opioid-sparing analgesia have also supported earlier mobilisation and recovery, with same-day mobilisation now routine [11, 43]. Similar trends have been reported internationally, albeit indirectly. In countries with healthcare systems comparable to the NHS, such as Denmark and Spain, reductions in LOS have occurred despite rising patient age and comorbidity [26, 39]. By contrast, in the USA, joint replacement surgery has shifted toward younger, healthier patients [44, 45]. This, along with perioperative pathway optimisation and an insurance-based funding model, may have contributed to the shorter LOS observed there. Accordingly, our findings show that procedures in the independent sector are strongly associated with shorter LOS. This is the first study to directly examine and quantify such associations in trends using a national dataset, uniquely incorporating patient, procedural, and socioeconomic characteristics.

The COVID-19 pandemic had a profound effect on joint replacement services in the UK, with almost a year’s worth of normal operating activity lost [7]. Substantial discrepancies in impact were observed across procedure types, countries, and provider sectors; from 2021 onwards, the independent sector became the predominant provider of joint replacements. This study identifies novel trends in patient characteristics during this period that reflect these inequities. Between 2020 and 2021, total hip and knee replacements were associated with sudden, significant decreases in patient age and ASA grade, and a non-significant reduction in obesity, likely reflecting a shift toward operating on lower-risk patients rather than changes in demand. By 2022, pre-pandemic demographic trends had resumed, suggesting higher-risk patients whose procedures were delayed may now face prolonged waiting times. Contrary to anecdotal reports of a step increase in patient frailty after the pandemic [46], these data suggest that long-term trends have continued unchanged in a linear fashion. Such reports are possibly explained by a perception of a sharp increase in frailty when normal services resumed after the period of relatively lower frailty during the pandemic.

Recovery projections suggest that it may take up to a decade to address the total joint replacement backlog [7]. The consequences of such prolonged delays are considerable, ranging from worsening pain, disability, and diminished quality of life at the individual level to increased healthcare usage and broader economic impact. In response, national strategies have focused on resource-efficient models of care, including the expansion of high-volume low-complexity (HVLC) surgical hubs [47] and the wider adoption of day case joint replacement pathways [13, 48]. The present results, where associations were demonstrated but LOS reduction has occurred independently despite characteristic trends and without an increase in readmission rates, are reassuring in suggesting that continued LOS reductions will be feasible despite the UK general population trends of increasing age, obesity, and comorbidity [14, 15].

However, these divergent trends should be carefully monitored, as reductions in LOS will likely plateau in a subset of high-risk patients who will continue to require inpatient care. Taken to extremes, there is a risk of widening healthcare inequalities and creating a two-tier system, where patients without the identified risk factors for prolonged LOS have greater access to surgery within expanding HVLC hubs and the independent sector, and patients with the identified risk factors are instead restricted to increasingly prolonged waiting times for traditional inpatient care. This will disproportionally affect older patients, those with multiple comorbidities, and the most socioeconomically deprived; groups already most severely affected by the pandemic [49]. Furthermore, characteristics associated with longer LOS such as obesity and multimorbidity are strongly correlated with socioeconomic deprivation [50], and without due care these trends therefore carry the risk of further worsening pre-existing inequalities reported in joint replacement provision [51].

### Limitations

The main limitation of this study is that although it represents a national sample of NHS-funded procedures, HES does not have coverage of independently funded procedures that occur in the independent sector. Although this was a relatively small proportion of cases (12.8% of NJR following exclusions in this dataset), the results are not necessarily generalisable to them. As independent sector provision was associated with shorter LOS, this may have biased overall results towards longer LOS. This limitation is likely to become increasingly relevant as independent sector activity continues to grow [7]. Future studies could address this by including data from independent hospital groups, or the Private Healthcare Information Network (PHIN), although no such source currently provides full national coverage [52].

Whilst multiple known factors were controlled for to evaluate the relationship between LOS and time specifically, there is potential for residual confounding from unmeasured characteristics. For instance, functional status is a recognised determinant of LOS but is not recorded in either HES or NJR datasets. However, as functional status will be associated with ASA grade, we expect this effect to be partially controlled for. Because many factors are likely to contribute to observed trends, it is not possible to quantify the specific impact of unmeasured / residual confounding. This indirect approach also limited the ability to identify which specific changes have had the greatest influence on trends, making it difficult to provide targeted recommendations for clinical practice.

Patient characteristics were modelled as linear associations, and although the disruptions around the pandemic appeared to resume previous linear trends, the study period ended in March 2022 when joint replacement provision had still not returned to pre-pandemic levels [7]. Ongoing monitoring is therefore required to assess whether these trends continue in a linear fashion once the recovery from the pandemic is complete. Finally, although the lack of increase in hospital readmission rates is reassuring and supports the safety and efficacy of efficiency measures to reduce LOS, this study did not assess potential increases in healthcare utilisation elsewhere, such as emergency department attendances without admission, primary care contacts, or increased burden on informal carers.

## Conclusions

Patients undergoing primary hip and knee replacement in England have seen a progressive reduction in LOS in hospital between 2010 and 2022. Patients can on average now expect to stay less than three days in hospital for a total hip or knee replacement, and less than two days for a partial knee replacement. These changes have occurred despite increasing patient comorbidity and obesity, suggesting that broader changes in perioperative care are the primary drivers. Reassuringly, this reduction in LOS has occurred without any concurrent increase in crude readmission rates.

These findings support the feasibility of ongoing national recovery efforts to expand high-volume surgical hubs and ambulatory joint replacement pathways. Continued LOS reductions appear achievable, even in the context of patient and population demographic trends. However, as LOS continues to decline, there is a risk of marginalising higher-risk patients who may still require traditional inpatient stays; these are the patients that were disproportionately affected during the pandemic. Ongoing monitoring will be essential to ensure that continued efficiency gains do not worsen health inequalities.

## Supplementary Information

Additional File 1: Figures S1–S6, Tables S1–S6. Figure S1 – Flowchart showing HES data cleaning rules. Figure S2 – Final fitted base flexible parametric survival models for each joint, with predicted survival visualised against crude Kaplan–Meier estimates. Figure S3 – Histograms showing length of stay distribution per procedure with median and mean indicated. Figure S4 – Association between age and median length of stay from multivariable model predictions. Figure S5 – Sensitivity analysis using univariable linear regression to estimate trends in mean length of stay. Figure S6 – Full Ovid search strategy and inclusion criteria for literature review. Table S1 – Characteristics of patients with and without IMD data. Table S2 – Observed yearly length of stay values per procedure. Table S3 – Trends in patient characteristics over time by procedure. Table S4 – Full multivariable flexible parametric model output with sensitivity analyses. Table S5 – Sensitivity analysis using linear regression with mean length of stay outcomes. Table S6 – Summary of identified studies and registry reports on length of stay.

## Data Availability

The datasets generated and analysed in the current study are not publicly available due to data protection regulations. Access to data is limited to the researchers who have obtained permission for data processing through the NJR Research Committee. Further inquiries can be made to the corresponding author.

## Abbreviations

AJRR: American Joint Replacement Registry
ASA: American Society of Anaesthesiologists
BIC: Bayesian Information Criterion
BMI: Body mass index
CI: Confidence interval
CJRR: Canadian Joint Replacement Registry
ERAS: Enhanced Recovery After Surgery
HES: Hospital Episode Statistics
HR: Hazard ratio
ICD: International Classification of Diseases
IMD: Index of Multiple Deprivation
IQR: Interquartile range
LOS: Length of stay
NHS: National Health Service
NJR: National Joint Registry
SAP: Scottish Arthroplasty Project
THR: Total hip replacement
TKR: Total knee replacement
UKR: Unicompartmental knee replacement
