# Supplementary material for: Trends in hip and knee replacement length of stay and patient demographics in England: a population-based study of 1,455,842 primary procedures
Source: BMC Med. 2025 Oct 14;23:561. doi: 10.1186/s12916-025-04294-6 (PMC12522415; doi:10.1186/s12916-025-04294-6)
Supplement: Supplementary file 1 — Additional File 1: Figures S1–S6, Tables S1–S6. Figure S1 – Flowchart showing HES data cleaning rules. Figure S2 – Final fitted base flexible parametric survival models for each joint, with predicted survival visualised against crude Kaplan–Meier estimates. Figure S3 – Histograms showing length of stay distribution per procedure with median and mean indicated. Figure S4 – Association between age and median length of stay from multivariable model predictions. Figure S5 – Sensitivity analysis using univariable linear regression to estimate trends in mean length of stay. Figure S6 – Full Ovid search strategy and inclusion criteria for literature review. Table S1 – Characteristics of patients with and without IMD data. Table S2 – Observed yearly length of stay values per procedure. Table S3 – Trends in patient characteristics over time by procedure. Table S4 – Full multivariable flexible parametric model output with sensitivity analyses. Table S5 – Sensitivity analysis using linear regression with mean length of stay outcomes. Table S6 – Summary of identified studies and registry reports on length of stay. [file 12916_2025_4294_MOESM1_ESM.docx]

Supplementary materials


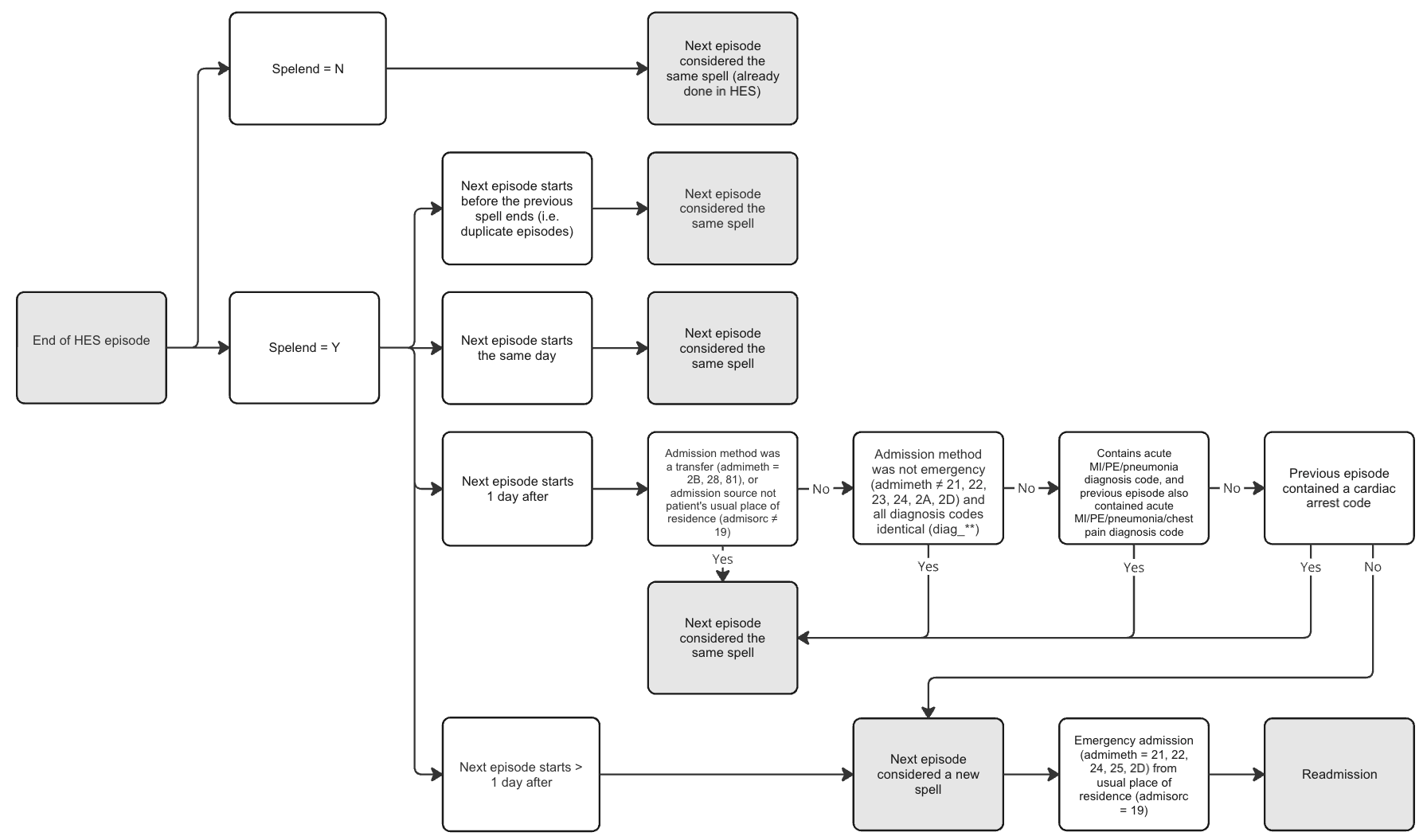


Figure S1 - Flowchart showing HES data cleaning rules.


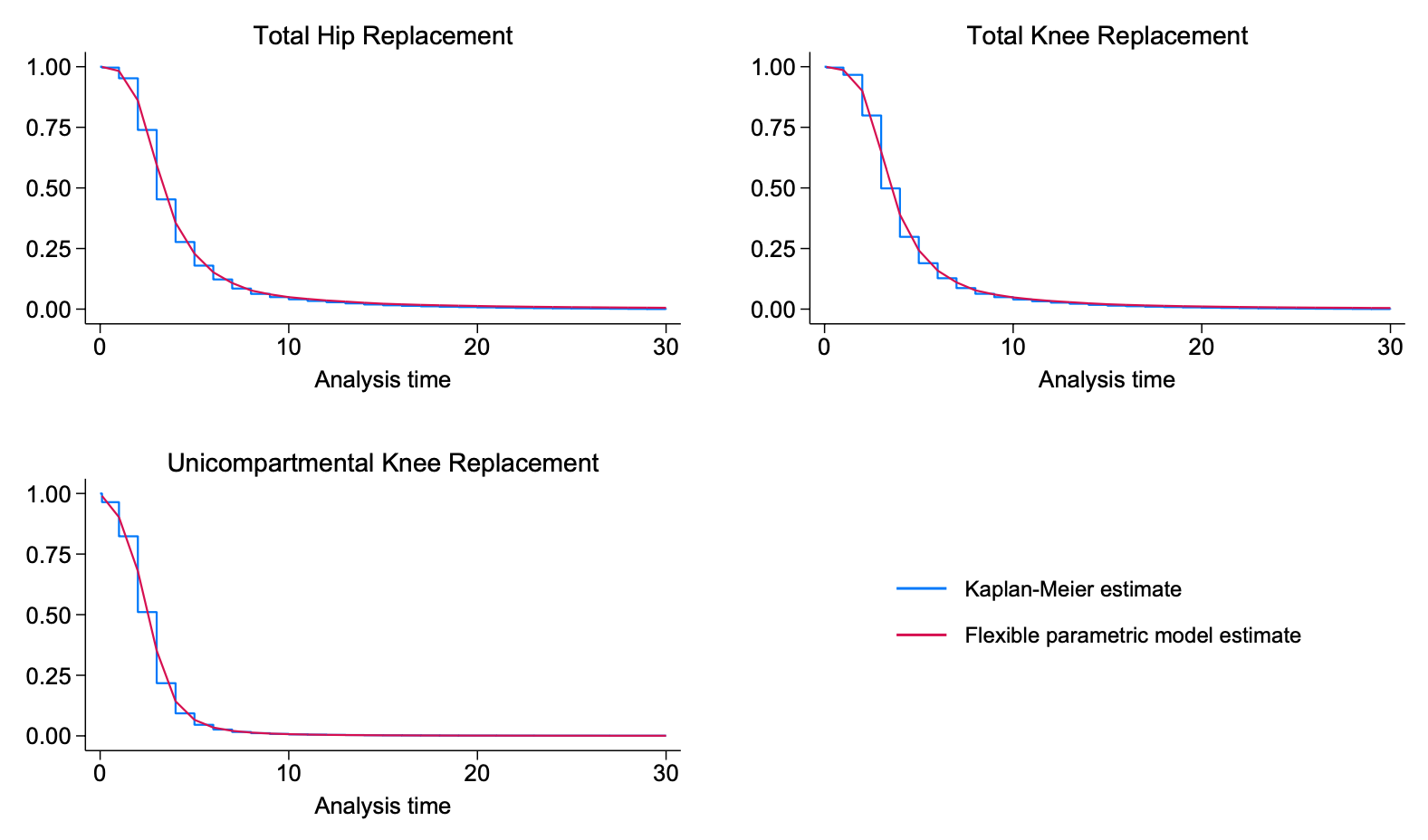


Figure S2 – Final fitted base flexible parametric survival models for each joint, with predicted survival visualised against crude Kaplan-Meier estimates. ‘Survival’ here represents time to hospital discharge.

|  | IMD available | IMD missing |
| --- | --- | --- |
|  | (N=1,445,189) | (N=10,653) |
| Age (years) | 68.75 (10.35) | 68.16 (10.69) |
| Sex |  |  |
| Female | 837,996 (58.0%) | 5,899 (55.4%) |
| Male | 607,193 (42.0%) | 4,754 (44.6%) |
| ASA grade |  |  |
| P1 - Fit and healthy | 145,666 (10.1%) | 1,296 (12.2%) |
| P2 - Mild disease not incapacitating | 1,029,483 (71.2%) | 7,743 (72.7%) |
| P3 - Incapacitating systemic disease | 264,419 (18.3%) | 1,591 (14.9%) |
| P4-5 - Life threatening disease | 5,621 (0.4%) | 23 (0.2%) |
| Obesity | 658,924 (45.6%) | 4,583 (43.0%) |
| Unit Sector |  |  |
| NHS | 1,007,198 (69.7%) | 10,327 (96.9%) |
| Independent | 437,991 (30.3%) | 326 (3.1%) |

Table S1
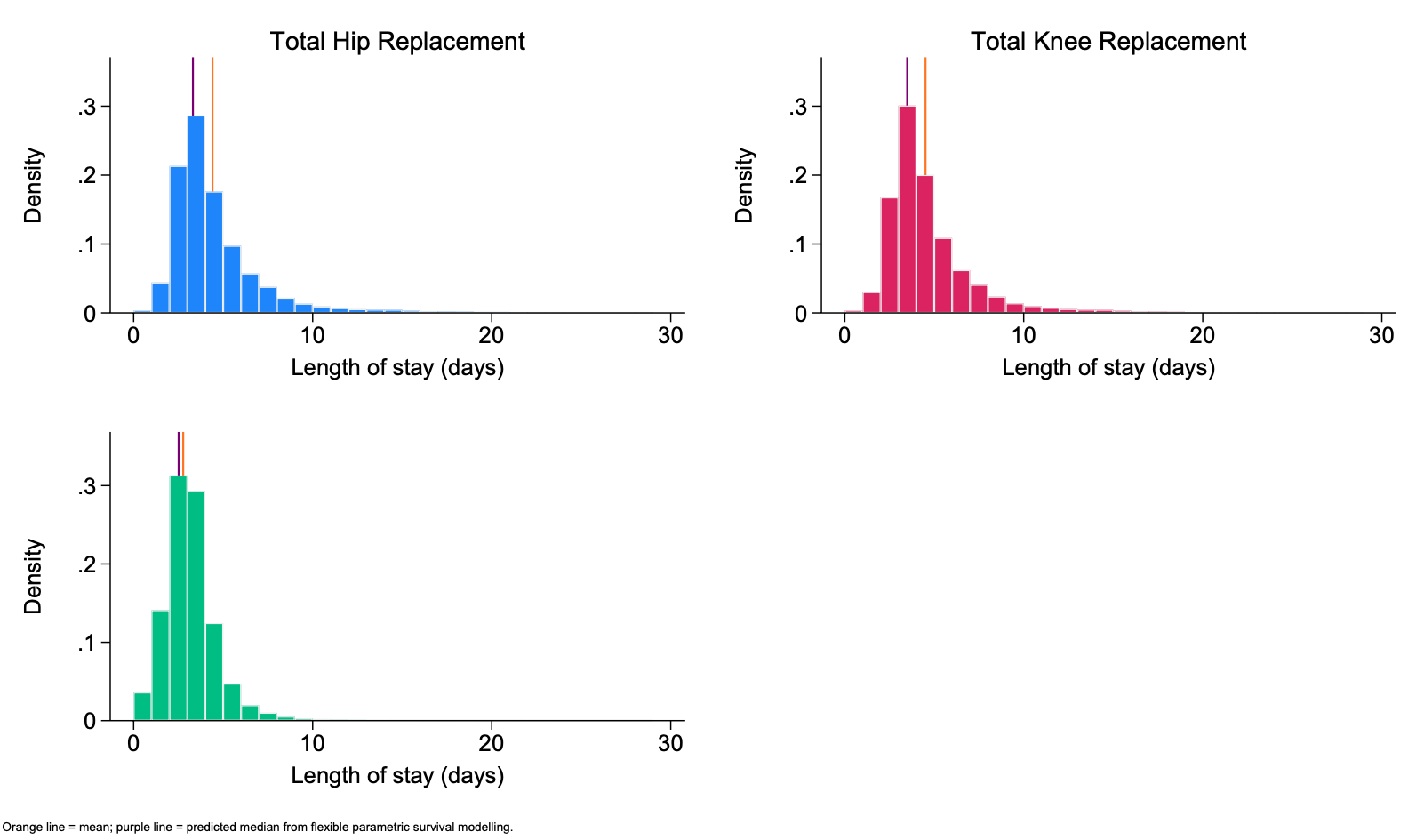
 - Table of patient characteristics between those with and without IMD data.

Figure S3 – Histograms showing length of stay distribution per procedure. The purple line represents estimated overall median from flexible parametric survival modelling. The orange line represents the mean (i.e. linear regression output).

| **Year** | **N** | **Mean** | **SD** | **Median** | **IQR** | | |
| --- | --- | --- | --- | --- | --- | --- | --- |
| *Total Hip Replacement* | | |  |  |  |  | |
| 2010 | 44966 | 6.02 | 6.35 | 5 | 4, | 6 |  |
| 2011 | 49109 | 5.56 | 8.26 | 4 | 3, | 6 |  |
| 2012 | 53300 | 5.28 | 8.37 | 4 | 3, | 6 |  |
| 2013 | 55602 | 4.91 | 5.54 | 4 | 3, | 5 |  |
| 2014 | 61177 | 4.64 | 5.29 | 4 | 3, | 5 |  |
| 2015 | 61954 | 4.48 | 4.85 | 3 | 3, | 5 |  |
| 2016 | 63268 | 4.36 | 5.47 | 3 | 3, | 5 |  |
| 2017 | 64730 | 4.05 | 4.32 | 3 | 2, | 4 |  |
| 2018 | 64099 | 3.81 | 4.02 | 3 | 2, | 4 |  |
| 2019 | 65634 | 3.61 | 4.07 | 3 | 2, | 4 |  |
| 2020 | 30083 | 3.20 | 3.31 | 3 | 2, | 3 |  |
| 2021 | 46878 | 3.14 | 3.95 | 2 | 2, | 3 |  |
| 2022 | 7815 | 2.89 | 3.03 | 2 | 2, | 3 |  |
| *Total Knee Replacement* | | |  |  |  |  | |
| 2010 | 49915 | 6.00 | 6.81 | 5 | 4, | 6 |  |
| 2011 | 53954 | 5.57 | 7.12 | 4 | 3, | 6 |  |
| 2012 | 57349 | 5.26 | 5.91 | 4 | 3, | 6 |  |
| 2013 | 59032 | 4.95 | 5.80 | 4 | 3, | 5 |  |
| 2014 | 66500 | 4.78 | 5.93 | 4 | 3, | 5 |  |
| 2015 | 68482 | 4.62 | 4.81 | 4 | 3, | 5 |  |
| 2016 | 70401 | 4.41 | 4.79 | 3 | 3, | 5 |  |
| 2017 | 71546 | 4.17 | 4.34 | 3 | 3, | 4 |  |
| 2018 | 67261 | 3.98 | 4.10 | 3 | 2, | 4 |  |
| 2019 | 70372 | 3.73 | 3.82 | 3 | 2, | 4 |  |
| 2020 | 29119 | 3.41 | 3.21 | 3 | 2, | 4 |  |
| 2021 | 44260 | 3.19 | 3.52 | 3 | 2, | 3 |  |
| 2022 | 7747 | 3.05 | 3.00 | 2 | 2, | 3 |  |
| *Unicompartmental Knee Replacement* | | | |  |  |  | |
| 2010 | 4117 | 3.74 | 2.36 | 3 | 3, | 4 |  |
| 2011 | 4281 | 3.59 | 2.28 | 3 | 2, | 4 |  |
| 2012 | 4976 | 3.36 | 2.54 | 3 | 2, | 4 |  |
| 2013 | 4942 | 3.17 | 2.09 | 3 | 2, | 4 |  |
| 2014 | 5841 | 3.03 | 1.97 | 3 | 2, | 4 |  |
| 2015 | 6210 | 2.94 | 2.11 | 3 | 2, | 3 |  |
| 2016 | 6754 | 2.85 | 1.75 | 3 | 2, | 3 |  |
| 2017 | 7551 | 2.70 | 1.92 | 3 | 2, | 3 |  |
| 2018 | 7576 | 2.51 | 2.16 | 2 | 2, | 3 |  |
| 2019 | 8240 | 2.28 | 1.69 | 2 | 1, | 3 |  |
| 2020 | 3920 | 1.99 | 1.83 | 2 | 1, | 3 |  |
| 2021 | 5902 | 1.86 | 1.53 | 2 | 1, | 2 |  |
| 2022 | 979 | 1.80 | 1.25 | 2 | 1, | 2 |  |

Table S2 - Observed yearly length of stay values per procedure.


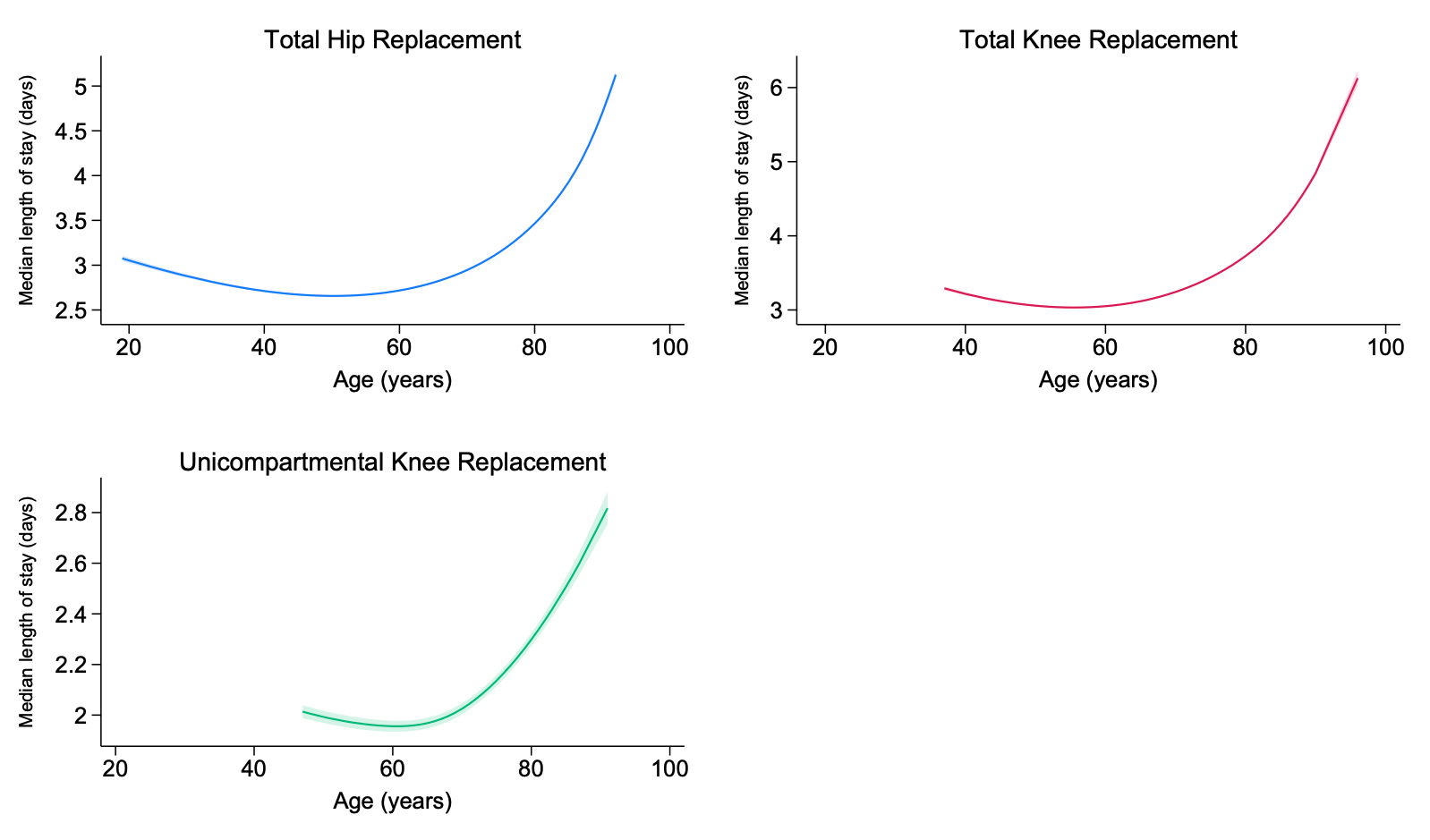


Figure S4 – Association between age and median length of stay, generated from multivariable flexible parametric model predictions for the average patient in 2021 with age modelled using restricted cubic splines. The shaded area represents 95% CI of estimates.

|  | **Total Hip Replacement** | | | |  | | **Total Knee Replacement** | | | | | |  | | **Unicompartmantal Knee Replacement** | | | | |
| --- | --- | --- | --- | --- | --- | --- | --- | --- | --- | --- | --- | --- | --- | --- | --- | --- | --- | --- | --- |
|  | Coef | 95% CI | | p-value | |  | | Coef | 95% CI | | p-value | | |  | | Coef | 95% CI | | p-value |
| Age | 0.00 | -0.01 | 0.01 | 0.803 | |  | | 0.01 | 0.00 | 0.01 | | 0.053 | |  | | 0.17 | 0.15 | 0.19 | 0.000 |
| Male (%) | 0.08 | 0.04 | 0.12 | 0.000 | |  | | 0.11 | 0.08 | 0.15 | | 0.000 | |  | | 0.36 | 0.25 | 0.47 | 0.000 |
| ASA ≥ 3 (%) | 0.75 | 0.72 | 0.78 | 0.000 | |  | | 0.70 | 0.67 | 0.72 | | 0.000 | |  | | 0.44 | 0.37 | 0.50 | 0.000 |
| Obesity (%) | 1.34 | 1.30 | 1.37 | 0.000 | |  | | 1.47 | 1.44 | 1.51 | | 0.000 | |  | | 1.29 | 1.18 | 1.40 | 0.000 |
| IMD quintile ≥ 3 (%) | -0.12 | -0.16 | -0.09 | 0.000 | |  | | -0.28 | -0.31 | -0.24 | | 0.000 | |  | | -0.34 | -0.46 | -0.23 | 0.000 |
| Independent sector (%) | 1.70 | 1.66 | 1.73 | 0.000 | |  | | 1.88 | 1.85 | 1.91 | | 0.000 | |  | | 1.76 | 1.65 | 1.86 | 0.000 |

Table S3 - Trends in patient characteristics over time for each procedure, from univariable linear regression models.

|  | **Total Hip Replacement**  **(HR (95%CI))** | | |  | **Total Knee Replacement**  **(HR (95%CI))** | | |  | **Unicompartmental Knee Replacement**  **(HR (95%CI))** | | |
| --- | --- | --- | --- | --- | --- | --- | --- | --- | --- | --- | --- |
|  | Unadjusted | Sensitivity* | Adjusted |  | Unadjusted | Sensitivity* | Adjusted |  | Unadjusted | Sensitivity* | Adjusted |
| Year | 1.089 | 1.089 | 1.106 |  | 1.087 | 1.087 | 1.096 |  | 1.105 | 1.105 | 1.111 |
|  | (1.088,1.090) | (1.088,1.090) | (1.105,1.107) |  | (1.086,1.088) | (1.086,1.088) | (1.096,1.097) |  | (1.102,1.108) | (1.103,1.108) | (1.108,1.113) |
| BH spline 1 | 3.455 | 3.455 | 3.893 |  | 3.439 | 3.439 | 3.794 |  | 3.575 | 3.575 | 3.693 |
|  | (3.445,3.466) | (3.445,3.465) | (3.882,3.905) |  | (3.429,3.449) | (3.429,3.449) | (3.783,3.805) |  | (3.538,3.612) | (3.538,3.612) | (3.655,3.732) |
| BH spline 2 | 1.256 | 1.256 | 1.211 |  | 1.184 | 1.184 | 1.149 |  | 0.786 | 0.786 | 0.762 |
|  | (1.252,1.260) | (1.252,1.260) | (1.207,1.215) |  | (1.180,1.188) | (1.180,1.188) | (1.145,1.152) |  | (0.781,0.791) | (0.781,0.791) | (0.758,0.767) |
| BH spline 3 | 1.436 | 1.436 | 1.447 |  | 1.466 | 1.467 | 1.474 |  | 1.205 | 1.204 | 1.192 |
|  | (1.433,1.439) | (1.433,1.439) | (1.444,1.450) |  | (1.463,1.469) | (1.464,1.470) | (1.471,1.477) |  | (1.199,1.210) | (1.199,1.209) | (1.187,1.197) |
| BH spline 4 | 1.040 | 1.040 | 1.065 |  | 1.094 | 1.094 | 1.115 |  | 1.132 | 1.131 | 1.139 |
|  | (1.037,1.042) | (1.037,1.042) | (1.063,1.068) |  | (1.092,1.097) | (1.092,1.097) | (1.113,1.118) |  | (1.128,1.136) | (1.128,1.135) | (1.135,1.143) |
| BH spline 5 | 0.957 | 0.957 | 0.976 |  | 0.950 | 0.950 | 0.969 |  | 1.004 | 1.004 | 1.011 |
|  | (0.955,0.959) | (0.955,0.958) | (0.974,0.977) |  | (0.948,0.951) | (0.948,0.951) | (0.967,0.970) |  | (1.001,1.007) | (1.001,1.007) | (1.009,1.014) |
| BH spline 6 | 0.968 | 0.968 | 0.970 |  | 0.964 | 0.964 | 0.963 |  | 0.986 | 0.986 | 0.986 |
|  | (0.967,0.969) | (0.967,0.969) | (0.969,0.971) |  | (0.963,0.965) | (0.963,0.965) | (0.962,0.964) |  | (0.984,0.988) | (0.984,0.988) | (0.984,0.988) |
| BH spline 7 | 0.992 | 0.992 | 0.994 |  | 0.992 | 0.992 | 0.993 |  | 0.998 | 0.998 | 0.998 |
|  | (0.992,0.993) | (0.992,0.993) | (0.993,0.995) |  | (0.991,0.992) | (0.991,0.992) | (0.992,0.994) |  | (0.997,0.999) | (0.997,0.999) | (0.997,0.999) |
| BH spline 8 | 0.992 | 0.992 | 0.991 |  | 0.993 | 0.993 | 0.992 |  | 0.998 | 0.998 | 0.998 |
|  | (0.991,0.992) | (0.991,0.992) | (0.990,0.991) |  | (0.993,0.994) | (0.993,0.994) | (0.991,0.992) |  | (0.997,0.999) | (0.997,0.999) | (0.997,0.999) |
| BH spline 9 | 0.995 | 0.995 | 0.995 |  | 0.996 | 0.996 | 0.996 |  |  |  |  |
|  | (0.995,0.996) | (0.995,0.996) | (0.994,0.995) |  | (0.996,0.996) | (0.996,0.996) | (0.995,0.996) |  |  |  |  |
| BH spline 10 | 0.996 | 0.996 | 0.995 |  | 0.996 | 0.996 | 0.996 |  |  |  |  |
|  | (0.996,0.996) | (0.996,0.996) | (0.995,0.995) |  | (0.996,0.997) | (0.996,0.997) | (0.995,0.996) |  |  |  |  |
| BH spline 11 | 1.000 | 1.000 | 1.000 |  | 1.000 | 1.000 | 1.001 |  |  |  |  |
|  | (1.000,1.000) | (1.000,1.000) | (1.000,1.000) |  | (1.000,1.001) | (1.000,1.001) | (1.000,1.001) |  |  |  |  |
| BH spline 12 | 1.000 | 1.000 | 1.000 |  | 1.000 | 1.000 | 1.000 |  |  |  |  |
|  | (0.999,1.000) | (0.999,1.000) | (1.000,1.000) |  | (1.000,1.000) | (1.000,1.000) | (1.000,1.000) |  |  |  |  |
| BH spline 13 | 1.000 | 1.000 | 1.001 |  | 1.001 | 1.001 | 1.001 |  |  |  |  |
|  | (1.000,1.001) | (1.000,1.001) | (1.000,1.001) |  | (1.000,1.001) | (1.000,1.001) | (1.001,1.001) |  |  |  |  |
| BH spline 14 | 1.000 | 1.000 | 1.000 |  | 1.000 | 1.000 | 1.000 |  |  |  |  |
|  | (1.000,1.000) | (1.000,1.000) | (1.000,1.000) |  | (1.000,1.000) | (1.000,1.000) | (1.000,1.000) |  |  |  |  |
| BH spline 15 | 1.000 | 1.000 | 1.000 |  | 1.000 | 1.000 | 1.000 |  |  |  |  |
|  | (1.000,1.000) | (1.000,1.000) | (1.000,1.000) |  | (1.000,1.000) | (1.000,1.000) | (1.000,1.000) |  |  |  |  |
| BH spline 16 | 0.999 | 0.999 | 0.999 |  | 1.000 | 1.000 | 0.999 |  |  |  |  |
|  | (0.999,0.999) | (0.999,0.999) | (0.999,0.999) |  | (0.999,1.000) | (0.999,1.000) | (0.999,1.000) |  |  |  |  |
| BH spline 17 | 1.000 | 1.000 | 1.000 |  | 1.000 | 1.000 | 1.000 |  |  |  |  |
|  | (1.000,1.000) | (1.000,1.000) | (0.999,1.000) |  | (1.000,1.000) | (1.000,1.000) | (1.000,1.000) |  |  |  |  |
| Age spline 1 |  |  | 0.773 |  |  |  | 0.795 |  |  |  | 0.911 |
|  |  |  | (0.771,0.775) |  |  |  | (0.793,0.797) |  |  |  | (0.904,0.919) |
| Age spline 2 |  |  | 1.178 |  |  |  | 1.125 |  |  |  | 1.107 |
|  |  |  | (1.175,1.181) |  |  |  | (1.122,1.128) |  |  |  | (1.099,1.115) |
| Age spline 3 |  |  | 1.017 |  |  |  | 1.003 |  |  |  | 1.031 |
|  |  |  | (1.015,1.020) |  |  |  | (1.001,1.006) |  |  |  | (1.024,1.039) |
|  |  |  | 1 |  |  |  | 1 |  |  |  | 1 |
|  |  |  | (ref) |  |  |  | (ref) |  |  |  | (ref) |
| Sex: Male |  |  | 1.200 |  |  |  | 1.130 |  |  |  | 1.210 |
|  |  |  | (1.194,1.206) |  |  |  | (1.125,1.136) |  |  |  | (1.192,1.228) |
| ASA 1 |  |  | 1 |  |  |  | 1 |  |  |  | 1 |
|  |  |  | (ref) |  |  |  | (ref) |  |  |  | (ref) |
| ASA 2 |  |  | 0.850 |  |  |  | 0.863 |  |  |  | 0.909 |
|  |  |  | (0.843,0.856) |  |  |  | (0.855,0.871) |  |  |  | (0.890,0.928) |
| ASA 3 |  |  | 0.544 |  |  |  | 0.592 |  |  |  | 0.652 |
|  |  |  | (0.538,0.549) |  |  |  | (0.586,0.598) |  |  |  | (0.632,0.673) |
| ASA 4-5 |  |  | 0.353 |  |  |  | 0.402 |  |  |  | 0.293 |
|  |  |  | (0.341,0.366) |  |  |  | (0.385,0.420) |  |  |  | (0.231,0.371) |
|  |  |  | 1 |  |  |  | 1 |  |  |  | 1 |
|  |  |  | (ref) |  |  |  | (ref) |  |  |  | (ref) |
| Obesity |  |  | 0.945 |  |  |  | 0.962 |  |  |  | 0.951 |
|  |  |  | (0.940,0.950) |  |  |  | (0.958,0.967) |  |  |  | (0.936,0.965) |
| IMD quintile 1 (least deprived) |  |  | 1 |  |  |  | 1 |  |  |  | 1 |
|  |  |  | (ref) |  |  |  | (ref) |  |  |  | (ref) |
| IMD quintile 2 |  |  | 1.002 |  |  |  | 1.003 |  |  |  | 1.008 |
|  |  |  | (0.995,1.009) |  |  |  | (0.996,1.010) |  |  |  | (0.987,1.029) |
| IMD quintile 3 |  |  | 0.983 |  |  |  | 0.977 |  |  |  | 0.988 |
|  |  |  | (0.976,0.990) |  |  |  | (0.970,0.984) |  |  |  | (0.968,1.010) |
| IMD quintile 4 |  |  | 0.936 |  |  |  | 0.945 |  |  |  | 0.950 |
|  |  |  | (0.929,0.943) |  |  |  | (0.938,0.952) |  |  |  | (0.928,0.972) |
| IMD quintile 5 (most deprived) |  |  | 0.871 |  |  |  | 0.911 |  |  |  | 0.909 |
|  |  |  | (0.864,0.879) |  |  |  | (0.904,0.918) |  |  |  | (0.885,0.933) |
|  |  |  | 1 |  |  |  | 1 |  |  |  | 1 |
|  |  |  | (ref) |  |  |  | (ref) |  |  |  | (ref) |
| Independent sector |  |  | 1.662 |  |  |  | 1.859 |  |  |  | 1.357 |
|  |  |  | (1.653,1.671) |  |  |  | (1.849,1.869) |  |  |  | (1.335,1.379) |
| N | 668511 | 662915 | 662915 |  | 715907 | 711309 | 711309 |  | 71289 | 70830 | 70830 |

Table S4 - Full multivariable flexible parametric model output, with sensitivity analysis where cases missing IMD data were excluded (*). HR = hazard ratio; CI = confidence intervals; BH = baseline hazard; spline = restricted cubic spline; ASA = American Society of Anaesthesiologists grade; IMD = index of multiple deprivation.


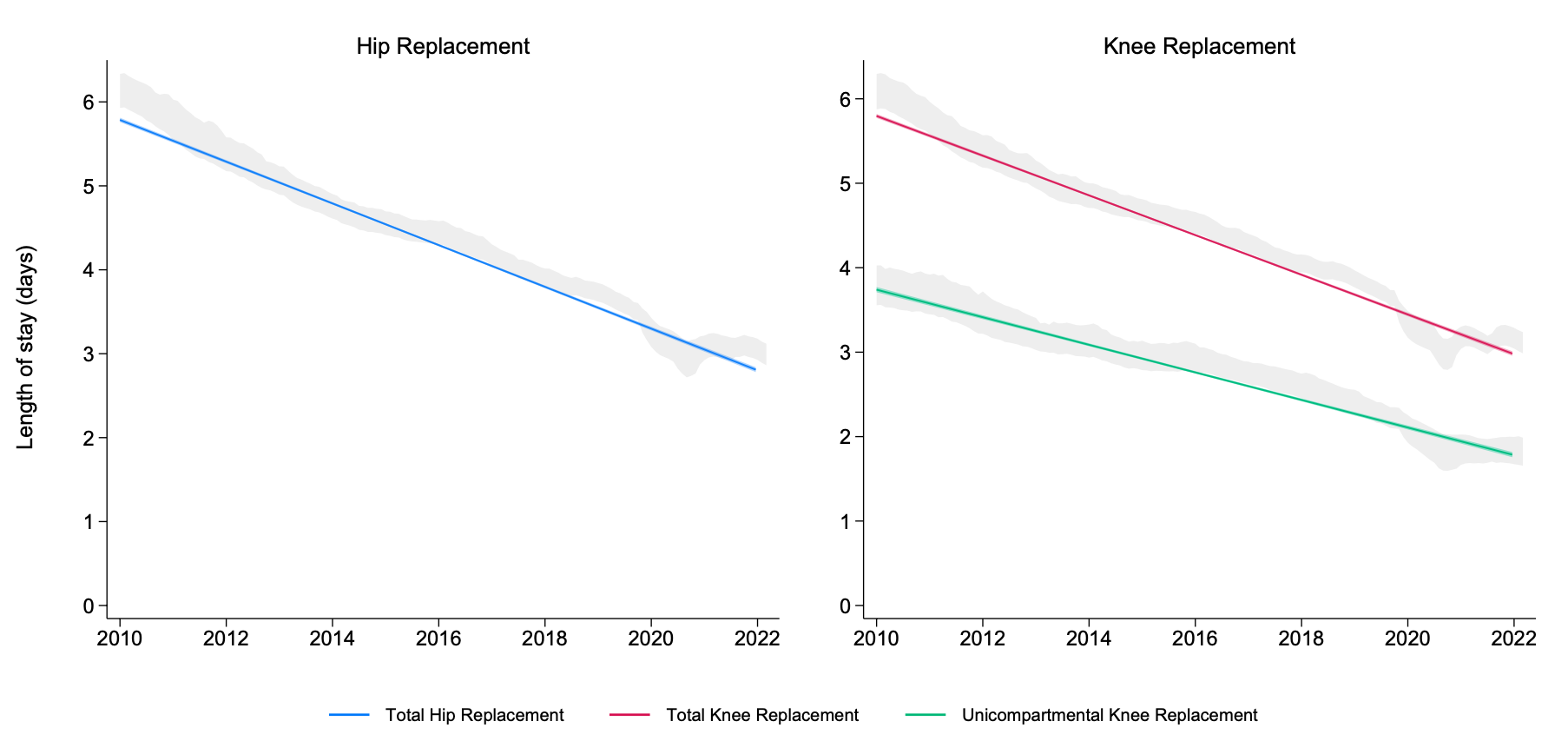


Figure S5 – Sensitivity analysis using univariable linear regression to estimate trends in mean length of stay, by procedure. Coloured shaded areas represent 95% CIs. The grey shaded area represents 95% CIs for the observed rolling monthly mean, centered over one year.

|  | **Total Hip Replacement**  **(Coefficient (95% CIs))** | |  | **Total Knee Replacement**  **(Coefficient (95% CIs))** | |  | **Unicompartmental Knee Replacement**  **(Coefficient (95% CIs))** | |
| --- | --- | --- | --- | --- | --- | --- | --- | --- |
|  | Unadjusted | Adjusted |  | Unadjusted | Adjusted |  | Unadjusted | Adjusted |
| Year | -0.248 | -0.246 |  | -0.234 | -0.221 |  | -0.163 | -0.164 |
|  | (-0.252,-0.244) | (-0.250,-0.242) |  | (-0.238,-0.231) | (-0.224,-0.217) |  | (-0.167,-0.158) | (-0.168,-0.160) |
| Age spline 1 |  | 0.871 |  |  | 0.784 |  |  | 0.178 |
|  |  | (0.857,0.884) |  |  | (0.772,0.796) |  |  | (0.163,0.193) |
| Age spline 2 |  | -0.680 |  |  | -0.502 |  |  | -0.192 |
|  |  | (-0.693,-0.667) |  |  | (-0.514,-0.490) |  |  | (-0.206,-0.177) |
| Age spline 3 |  | -0.357 |  |  | -0.202 |  |  | -0.0965 |
|  |  | (-0.370,-0.345) |  |  | (-0.214,-0.191) |  |  | (-0.111,-0.0822) |
| Sex: Male |  | -0.410 |  |  | -0.232 |  |  | -0.296 |
|  |  | (-0.436,-0.384) |  |  | (-0.256,-0.209) |  |  | (-0.325,-0.267) |
| ASA 1 |  | 0 |  |  | 0 |  |  | 0 |
|  |  | (ref) |  |  | (ref) |  |  | (ref) |
| ASA 2 |  | 0.307 |  |  | 0.291 |  |  | 0.145 |
|  |  | (0.265,0.348) |  |  | (0.246,0.335) |  |  | (0.104,0.185) |
| ASA 3 |  | 1.867 |  |  | 1.631 |  |  | 0.798 |
|  |  | (1.816,1.918) |  |  | (1.579,1.682) |  |  | (0.737,0.858) |
| ASA 4-5 |  | 4.811 |  |  | 4.487 |  |  | 3.774 |
|  |  | (4.625,4.996) |  |  | (4.278,4.696) |  |  | (3.319,4.229) |
| Obesity |  | 0.0997 |  |  | 0.0498 |  |  | 0.0552 |
|  |  | (0.0728,0.127) |  |  | (0.0256,0.0739) |  |  | (0.0255,0.0850) |
| IMD quintile 1 (least deprived) |  | 0 |  |  | 0 |  |  | 0 |
|  |  | (ref) |  |  | (ref) |  |  | (ref) |
| IMD quintile 2 |  | 0.0222 |  |  | -0.00717 |  |  | 0.0163 |
|  |  | (-0.0140,0.0584) |  |  | (-0.0416,0.0273) |  |  | (-0.0240,0.0567) |
| IMD quintile 3 |  | 0.0815 |  |  | 0.0825 |  |  | 0.0340 |
|  |  | (0.0442,0.119) |  |  | (0.0475,0.117) |  |  | (-0.00750,0.0756) |
| IMD quintile 4 |  | 0.219 |  |  | 0.155 |  |  | 0.0964 |
|  |  | (0.178,0.259) |  |  | (0.119,0.192) |  |  | (0.0509,0.142) |
| IMD quintile 5 (most deprived) |  | 0.387 |  |  | 0.287 |  |  | 0.160 |
|  |  | (0.342,0.432) |  |  | (0.248,0.327) |  |  | (0.109,0.212) |
| Independent sector |  | -1.037 |  |  | -1.206 |  |  | -0.301 |
|  |  | -1.065,-1.008 |  |  | -1.232,-1.180 |  |  | -0.332,-0.270 |

Table S5 - Sensitivity analysis: unadjusted and adjusted multivariable linear regression giving mean rather than median values to determine the extent to which trends could be explained by changes in patient characteristics. Note age had a non-linear association with length of stay and therefore individual coefficients for each spline cannot be directly interpreted, see Supplementary Figure 6. CI = confidence interval; spline = restricted cubic spline; ASA = American Society of Anaesthesiologists grade; IMD = index of multiple deprivation.

Figure S6 – Full Ovid search strategy. Inclusion criteria were studies on primary hip and knee procedures which included a measure of length of stay. Studies had to be non-selective i.e. single-centre studies, or multi-centre studies of specifically “fast-track” or day case centres, were excluded. Where multiple studies on the same database or country were present, the largest, least selective, and most up to date study was included.

Ovid MEDLINE(R) <1946 to present>

Embase <1974 to 2024 October 24>

1 ("hip replacement" or "knee replacement" or "hip arthroplasty" or "knee arthroplasty" or "THR" or "TKR" or "UKR" or "THA" or "TKA" or "UKA").ti. 121342

2 ("length of stay" or "hospital stay" or "inpatient stay" or "hospitalized duration" or "inpatient duration").ti. 24358

3 1 and 2 852

4 remove duplicates from 3 518

| **Study** | **Country** | **Database** | **Joint** | **Cases (n=)** | **Year(s)** | **LOS (mean)** | **SD** | **LOS (median)** |
| --- | --- | --- | --- | --- | --- | --- | --- | --- |
| American Joint Replacement Registry Annual Report (2023) | US | AJRR | THR | 752,866 | 2010 to 2022 | 3.0 to 1.2 |  |  |
|  |  |  | TKR | 1,151,042 | 2010 to 2022 | 2.9 to 1.2 |  |  |
|  |  |  | UKR |  | 2010 to 2022 | 2.3 to 0.6 |  |  |
| Venishetty (2023) | US | National Inpatient Sample | THR | 367,890 | 2016 to 2019 | 2.06 | 1.89 |  |
| Laver (2024) | US | National Inpatient Sample | TKR | 2,636,880 | 2016 to 2019 | 2.28 |  |  |
| Scottish Arthroplasty Project Annual Report (2024) | Scotland | SAP | THR |  | 2010 to 2023 | 6.32 to 2.74 |  | 5 to 2 |
|  |  |  | KR |  | 2010 to 2023 | 6.22 to 2.92 |  | 5 to 2 |
| Canadian Joint Replacement Registry Annual Report (2022) | Canada | CJRR | THR | 58,635 | 2021 | 2.3 |  | 2 |
|  |  |  | KR | 58,443 | 2021 | 2.3 |  | 2 |
| Jensen (2023) | Denmark | Danish Inpatient Register | THR | 86,070 | 2010 to 2020 |  |  | 3 to 1 |
|  |  |  | TKR | 70,323 | 2010 to 2020 |  |  | 3 to 1 |
|  |  |  | UKR | 10,440 | 2010 to 2020 |  |  | 2 to 1 |
| Pamilo (2013) | Finland | Hospital Discharge Register | THR | 54,505 | 1998 to 2010 | ~10 to ~5 |  |  |
| Jenny (2021) | France | French agency for information on hospital care (ATIH) | TKR | 96,329 | 2017 | 6.4 | 3.1 |  |
| De Ladoucette (2020) | France | French agency for information on hospital care (ATIH) | THR | 104,745 | 2016 | 6.3 | 2.9 |  |
| Langenberger (2023) | Germany | PROMoting Quality study | THR | 2,611 | 2019 to 2020 | 6.6 | 2.95 |  |
|  |  |  | KR | 2,077 | 2019 to 2020 | 6.41 | 2.14 |  |
| Burn (2018) | England | Clinical Practice Research Datalink and Hospital Episode Statistics | THR | 10,961 | 1997 to 2014 | 14.4 to 5.6 |  |  |
|  |  |  | TKR | 10,260 | 1997 to 2014 | 16 to 5.4 |  |  |
| Oldmeadow (2002) | Australia | 3 hospitals | TKR | 105 | 1999 | 6.5 |  |  |
| Olthof (2017) | Netherlands | 3 hospitals | THR | 2,282 | 2009 to 2013 | 8.4 | 5.7 |  |
| Long (2023) | China | Hospital Quality Monitoring System | TKR | 184,363 | 2013 to 2019 | 10.8 to 9.3 |  |  |
| Chou (2019) | Taiwan | National Health Insurance Research Database | THR | 6,367 | 2012 | 7.3 | 4.3 |  |
| Hara (2024) | Japan | Diagnosis Procedure Combination database | TKR | 5,831 | 2018 to 2022 |  |  | 21 (18 to 26) |
| Jimenez-Garcia (2011) | Spain | National Hospital Discharge Database | THR | 161,791 | 2001 to 2008 | 13 to 10.45 |  | 11 to 8 |
| Chevalier (2015) | Belgium | IMS Hospital Disease Database | THR | 7,160 | 2013 | 13.59 |  |  |
|  |  |  | TKR | 6,223 | 2013 | 9.9 |  |  |
| Lao (2019) | New Zealand | National Minimum Dataset | THR | 53,439 | 2005 to 2017 | 6.2 to 3.8 | 3.2 to 2.4 | 6 to 3 |
|  |  |  | TKR | 50,072 | 2005 to 2017 | 6.6 to 4.1 | 3.5 to 2.1 | 6 to 4 |

Table S6 - Summary of identified studies and registry reports on length of stay (8, 22-39).
